# Variability in the Follow-up Management of Pediatric Femoral Fractures

**DOI:** 10.5435/JAAOSGlobal-D-20-00084

**Published:** 2022-04-26

**Authors:** Gabrielle E. Sanatani, Eva Habib, Jeffrey N. Bone, Ash Sandhu, Emily K. Schaeffer, Kishore Mulpuri

**Affiliations:** From the Royal College of Surgeons in Ireland, Dublin, Ireland (Sanatani); the Department of Orthopaedic Surgery, British Columbia Children's Hospital, Vancouver, British Columbia, Canada (Habib, Dr. Schaeffer, and Dr. Mulpuri); the British Columbia Children's Hospital Research Institute, Vancouver, British Columbia, Canada (Bone); and the Department of Obstetrics and Gynaecology, University of British Columbia, Vancouver, British Columbia, Canada (Sandhu).

## Abstract

**Introduction:**

Variability in follow-up has previously been identified in orthopaedic trauma. Variability in follow-up for pediatric femur fractures has not previously been documented. The aim of this study was to document the variability in clinical and radiographic follow-up for pediatric femur fractures based on the fixation method and the treating surgeon.

**Methods:**

This retrospective case series identified isolated femoral fractures in patients younger than 18 years, treated by eight surgeons at a single center from 2010 to 2015. The total number and frequency of clinical visits, radiographic visits and discrete radiograph views, demographic data, fracture classification, treatment method, and presence of complications were extracted. Variability in follow-up was assessed through descriptive statistics and linear and Poisson regression models.

**Results:**

One hundred sixty-four femoral fractures in 160 patients were included. Fractures were stratified by the treating surgeon. The mean length of follow-up ranged from 6.5 to 13.6 months. Complications increased follow-up time by mean 1.7 months (1.3 to 2.4). Patients who were treated with rigid locking nails were followed for the shortest amount of time, averaging 9.9 months, while traction followed by rigid locking nails averaged 24.4 (0.5 to 9.3) months of follow-up.

**Discussion:**

Variation in the length of follow-up was identified and was associated with the fixation method and the treating surgeon. Few patients were followed long enough to definitively identify complications and sequelae known to occur after femur fractures such as femoral overgrowth or growth arrest. The results of this study indicate a need for additional study and consensus on an appropriate follow-up for pediatric femur fractures.

Femoral fractures represent the most common orthopaedic injury requiring hospitalization in the pediatric population, comprising approximately 20% of these injuries.^1,2,3,4^ The incidence of diaphyseal femoral fractures is estimated to be 19 fractures per 100,000 children annually.^1^ The most common treatment options include hip spica casting, traction, elastic stable intramedullary nailing, rigid locking nail, and plating.^5^ In children younger than 6 months , a Pavlik harness can also be a viable treatment option.^6^ It is well known that patients' age, size, and fracture pattern will influence treatment, but surgeon preference may also play a role, particularly in clinical follow-up practices.^7^

The American Academy of Orthopaedic Surgeons has published guidelines outlining treatment practices for pediatric diaphyseal femur fractures, but these guidelines lack comprehensive suggestions on the ideal duration or frequency of follow-up.^8,9^ Most current published literature assesses one or two treatment types or evaluates treatment practices for one specific type of femur fracture. The duration and frequency of follow-up are not rigorously evaluated and can often be due to surgeon preference.^10^

A recent study by Adamich et al examined the necessity for the orthopaedic follow-up in the management of pediatric clavicle fractures, focusing on when the decision is made to treat patients surgically. This led to the conclusion that not all clavicle fractures require an orthopaedic follow-up.^11^ Applying a similar methodology, we assessed the duration and frequency of orthopaedic follow-up in pediatric femur fractures because this has not previously been evaluated. The primary purpose of this study was to assess variability in follow-up practices in pediatric femur fractures between surgeons at a single center adjusting for patient age and fracture type.

## Methods

After obtaining ethics approval from the university institution-affiliated research ethics board, a retrospective chart review was conducted for all patients presenting to a single tertiary pediatric care center who sustained an isolated femoral fracture between January 1, 2010, and December 31, 2015. Surveillance for follow-up visits was conducted through December 31, 2019. Over this period, there were eight pediatric orthopaedic surgeons managing femur fractures at this center. Patients were identified from a surgical database and from the hospital's Performance Measurement and Reporting Agency using appropriate diagnosis and procedure codes (Table 1).^12,13^ Operations missing procedure codes were identified using the diagnosis. Key diagnosis search terms included femur #, femur fracture, femoral fracture, # right femur, # left femur, # femur, fracture femur, fracture left femur, fracture right femur, bilateral femur fracture, femoral shaft fracture, femur shaft fracture, femur shaft #, hip fracture, and femur malunion. Medical records and radiographs of potentially eligible patients were reviewed to confirm inclusion.

**Table 1:** ICD-10^12^ Diagnosis Codes and CPT^13^ Procedure Codes Used to Identify Femur Fracture Treatment and Treatment of Complications

Codes Used to Identify Fracture Treatment and Diagnosis	
CPT procedure codes	
80510	Application cast spica hip
81505	Change pin external fixator
83401	Insertion-pin-femur/tibia-traction
84810	Manipulation and cast
86510	Reduction-fracture-femur-neck-pinning
86520	Reduction-fracture-femur-subtrochanteric-open/DHS
86530	Reduction-fracture-femur-shaft-open/rod
86531	Reduction-fracture-femur-shaft-open/plate and screws
86532	Reduction-fracture-femur-shaft-percutaneous/rod
86533	Reduction-fracture-femur-shaft-percutaneous/nancy nail
86535	Reduction-fracture-femur-closed
86540	Reduction-fracture-femur-supracondylar-open/plate and screws
ICD-10 diagnostic codes	
S72.000	Fracture of upper femoral epiphysis (separation), closed
S72.010	Fracture of base of femoral neck (cervicotrochanteric), closed
S72.080	Other fracture of femoral neck, closed
S72.090	Unspecified fracture of neck of femur, closed
S72.100	Intertrochanteric fracture, closed
S72.190	Unspecified trochanteric fracture, closed
S72.200	Subtrochanteric fracture, closed
S72.300	Fracture of shaft of femur, closed
S72.400	Fracture of lower femoral epiphysis (separation), closed
S72.410	Condylar fracture of femur, closed
S72.420	Supracondylar fracture of femur, closed
S72.490	Unspecified fracture of lower (distal) end of femur, closed
S72.800	Fractures of other parts of femur, closed
S72.900	Fracture of femur, part unspecified, closed
Codes used to identify complications	
87205	Removal-nail-nancy
87213	Removal-plate & screws-femur

ICD-10 = The International Classification of Disease 10, CPT = Current Procedural Terminology, DHS = dynamic hip screw.

Patients were included if they were younger than 18 years at presentation and were treated for an isolated femur fracture. Patients were excluded if there were any pre-existing pathologies increasing the likelihood of a fracture (eg, osteogenesis imperfecta) or history of a previous femur fracture at the same site. Patients were also excluded if they presented with open fractures or fractures at other sites of the body, with the exception of bilateral femur fractures. Open fractures were excluded because the treatment and potential for complications vary greatly from those of closed fractures. Patients were excluded if they were lost to follow-up at any point during the duration of their care. This was determined by a lack of documented clinic or radiographic visits, despite a clinic note saying the patient is due to return. The presence of complications was documented for all clinic visits. Cast irritation requiring early removal, growth arrest, infection, notable pain, refracture, and nonunion were analyzed as true complications. Implant irritation and leg-length discrepancy were analyzed as expected sequelae.

The primary outcome was the duration of clinical follow-up. The secondary outcome was the frequency of clinical follow-up, defined as discrete visits to the orthopaedic clinic. The number of radiographs included all discrete views obtained of the fracture site. Specifically, the authors were interested in assessing duration and frequency when patients were stratified according to the treating surgeon. Demographic data, such as sex and age, and injury characteristics, including injury mechanism and side of injury, were collected. Initial treatment, including emergency department presentation and treatment, and surgical details for all surgeries were collected. Follow-up data were collected for every clinic visit until the patient was discharged from care at the hospital. Radiographic images and reports were analyzed to determine fracture location, classification, and epiphyseal plate status. One medical student was responsible for extracting all data from patient charts, and radiographic images were analyzed by one pediatric orthopaedic fellow. Fractures were classified according to the Arbeitsgemeinschaft für Osteosynthesefragen classification of long bone fracture guidelines.^9^

All data were collected from hospital electronic health records using the Electronic Viewer for Everyone, PowerChart, and the Picture Archiving and Communication System. All collected information was deidentified to maintain patient confidentiality, and all data were stored and managed using Research Electronic Data Capture hosted at the affiliated institution.^14,15^ Research Electronic Data Capture is a secure, web-based software platform designed to support data capture for research studies (Vanderbilt University).

Patient demographics and clinical characteristics were summarized with counts and percentages. We summarized the mean follow-up time (in months), the mean number of follow-ups, radiographs and radiographic visits by the surgeon, and surgery type. To compute adjusted estimates and 95% confidence intervals, we used linear regression models for follow-up time and negative binomial models for the remaining outcomes. For linear models, we report the adjusted mean difference between surgeons, and for negative binomial models, we report rate ratios. All models were adjusted for age, sex, complications, and fracture type, which were identified a priori because important clinical factors for the outcome that would likely vary between both surgeons and surgery types. Negative binomial models included an offset term for time (in weeks). Based on our sample size (N = 160), we determined that models including 5 to 10 terms such as these were reasonable based on previously published guidance for regression modeling.^16^ To test for overall differences in outcomes by surgeon and surgery type, we used the likelihood ratio test to compare models with and without surgeon/surgery type. This test determines whether the inclusion of a variable explains markedly more of the variance in the outcome compared with a simpler model (ie, including just the adjustment factors above). We did not compute all pairwise *P*-values between surgeons and surgery types because these hypotheses were not of primary interest. We did not have sufficient data to study subgroup (ie, interactions between surgeon and/or surgery type and age, sex, etc.) differences. We also found that models including both surgeon and surgery type led to unstable fits (because of collinearity), and therefore, we kept the analyses separate to ensure meaningful estimates. All analyses were done using R statistical software, version 3.5.3, and a significance level was set at 0.05.^17^

## Results

**Figure 1:**
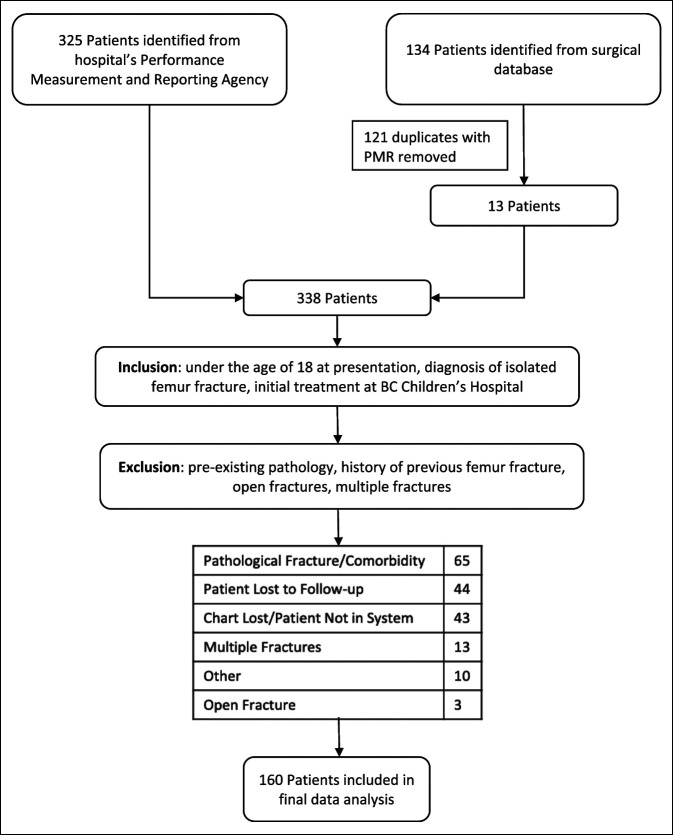
Flow chart describing the inclusion process for patients included in cohort.

Three hundred twenty-five patients were identified using the hospital's Performance Measurement and Reporting Agency while 134 were identified from the orthopaedic clinic’s surgical database (Figure 1). From there, 121 duplicates were removed, and the charts and radiographs of these 338 potentially eligible patients were then reviewed to identify 160 total patients who met the inclusion criteria. Of these 160 patients, 164 fractures were identified when accounting for bilateral femur fractures. The makeup of excluded patients is shown in Figure 1.

The table and following results include patients who received the entirety of their care at our institution and for whom full follow-up data were ascertained. The mean age of the patient cohort was 4.4 years, and 118 men and 42 women were identified (Table 2). Sixty-seven patients had a right femoral fracture, 90 had a left femoral fracture, and three had bilateral femoral fractures. One bilateral patient was identified as having two separate fractures on their left femur and was therefore counted as having three femur fractures in this study. In most cases (75.6%), patients presented to external hospitals or clinics before presenting to our institution for definitive treatment. The incidence of fracture types is given in Table 2.

**Table 2:** Patient Demographics, Fracture Characteristics, Treatment Details, and Documented Complications

Factor	Overall (n [%])
n	164
Male sex (%)	118 (160 [73.8])
Side injured (%)	
Bilateral	7 (4.3)
Left	90 (54.9)
Right	67 (40.9)
Femur fracture type (%)	
31-E proximal epiphyseal fracture	1 (0.6)
31-M proximal metaphyseal fracture	9 (5.5)
32-D diaphyseal fracture	145 (88.4)
33-E distal epiphyseal fracture	2 (1.2)
33-M distal metaphyseal fracture	7 (4.3)
Fracture location (%)	
Distal third	16 (9.8)
Distal two-thirds	16 (9.8)
Middle third	75 (45.7)
Proximal third	29 (17.7)
Proximal two-thirds	28 (17.1)
Initial treatment at this center's ER? (%)	
No	124 (75.6)
Unknown	12 (7.3)
Yes	28 (17.1)
Age at injury (mean [SD])	4.41 yr (4.5)
Treating surgeon (%)	
A	25 (15.2)
B	41 (25.0)
C	33 (20.1)
D	4 (2.4)
E	32 (19.5)
F	13 (7.9)
G	7 (4.3)
H	9 (5.5)
Treatment type (%)	
Elastic stable nailing (ESIN)	30 (18.3)
Other	16 (9.8)
Traction and spica cast	44 (26.8)
Plating	3 (1.8)
Rigid locking nail	13 (7.9)
Spica cast	48 (29.3)
Traction, conventional locking nail	3 (1.8)
Traction, elastic stable nailing (ESIN)	7 (4.3)
Complication documented (n = 13) (%)	
Cast irritation	3 (23.0)
Growth arrest	1 (7.7)
Infection	3 (23.0)
Other	3 (23.0)
Significant pain	3 (23.0)

The value of n is adjusted for fracture number or patient number accordingly. ER = emergency room.

Surgeons A and E followed patients for the shortest and longest duration of time, averaging 6.5 months and 13.6 months, respectively. We found that the surgeon was an independent predictor of mean duration time (*P* = 0.03) with differences mostly varying from 1 to 2 months in average follow-up time after adjusting for other relevant variables (see Methods). Similar differences were observed in follow-up appointments, radiographic visits, and the number of radiographs obtained where an adjusted relative number of outcomes between surgeons varied from 20% to 40% (Table 3).

**Table 3:** Regression Summary by Treating Surgeon

Treating Surgeon	Mean Follow-up Times in mo	Mean Difference (95% CI)^a^	Mean No. of Follow-ups	Relative Rate (95% CI)^a^	Mean No. of x-rays	Relative Rate (95% CI)^a^	Mean No. of Radiographic Visits	Relative Rate (95% CI)^a^
A (n = 25)	6.5	Reference	3.9	Reference	17.2	Reference	7.2	Reference
B (n = 41)	12.6	1.5 (1.0-2.5)	3.7	0.8 (0.6-1.1)	16.4	0.8 (0.7-1.0)	6.9	0.9 (0.7-1.1)
C (n = 33)	8.6	1.2 (0.8-2.0)	3.4	0.9 (0.7-1.2)	15.8	0.9 (0.7-1.1)	7.16	1.0 (0.8-1.21)
D (n = 4)	10.5	1.1 (0.4-2.9)	3.8	0.8 (0.4-1.3)	19.0	0.8 (0.6-1.2)	6.3	0.7 (0.5-1.1)
E (n = 32)	13.6	2.0 (1.2-3.2)	3.8	0.9 (0.7-1.2)	16.1	1.0 (0.8-1.1)	7.6	1.0 (0.8-1.3)
F (n = 13)	8.0	0.8 (0.4-1.4)	3.8	0.7 (0.5-1.1)	19.5	0.9 (0.7-1.1)	8.1	0.9 (0.7-1.2)
G (n = 7)	8.4	0.9 (0.4-2.0)	4.0	1.0 (0.7-1.6)	15.1	0.9 (0.7-1.2)	7.3	1.0 (0.7-1.4)
H (n = 9)	10.7	1.3 (0.6-2.5)	4.2	1.2 (0.8-1.7)	14.1	1.0 (0.7-1.3)	6.8	1.0 (0.7-1.4)
*P*-value^b^	0.03		0.002		<0.001		0.01	

CI = confidence interval

a Adjusted for age, sex, complications, and fracture type. Mean difference estimated from linear regression models and relative rates estimated from negative binomial models.

b Calculated from the likelihood ratio test comparing models including and not including treating surgeon.

Patients treated with rigid locking nails were followed for the shortest amount of time, averaging 9.9 months (Table 4). Patients who were treated with a combination of skeletal traction and rigid locking nails were followed for the longest amount of time, averaging more than 2 years in the follow-up. However, including treatment type in a model explaining variation in outcome did not improve the fit (*P* = 0.18), indicating that these differences may be explained by other factors. The treatment type was also found to not play a significant role in determining the total number of follow-up appointments (*P* = 0.24). However, the treatment type did help explain (*P* < 0.001) differences in the rate of radiographs obtained for the fracture site and in the number of radiographic visits (*P* < 0.001). Importantly, the analyses assessing surgeon variability and treatment differences may be in part measuring the same thing.

**Table 4:** Regression Summary by Treatment Type

Surgery	Mean Follow-up Times in mo	Mean Difference (95% CI)^a^	Mean No. of Follow-ups	Relative Rate (95% CI)^a^	Mean No. of x-rays	Relative Rate (95% CI)^a^	Mean No. of Radiographic Visits	Relative Rate (95% CI)^a^
Rigid locking nail (n = 13)	9.9	Reference	4.5	Reference	26.8	Reference	7.9	Reference
Elastic stable nailing (ESIN) (n = 30)	11.6	2.1 (0.9-4.8)	4.6	1.5 (1.0-2.6)	20.4	1.4 (1.1-1.8)	8.2	1.6 (1.2-2.3)
Other (n = 16)	9.7	1.8 (0.6-5.3)	3.5	1.5 (0.9-2.8)	14.4	1.1 (0.8-1.6)	6.4	1.5 (1.0-2.3)
Traction and spica cast (n = 44)	11.8	2.6 (1.0-6.8)	3.8	1.7 (1.0-2.8)	17.8	1.8 (1.3-2.3)	8.5	2.4 (1.7-3.5)
Plating (n = 3)	13.9	1.8 (0.2-11.0)	6.0	1.3 (0.5-3.5)	38.0	1.8 (1.0-3.0)	10.0	1.4 (0.7-2.7)
Spica cast (n = 48)	7.2	1.8 (0.6-4.9)	2.9	1.6 (0.9-2.7)	11.3	1.3 (2.0-1.8)	5.5	1.8 (1.2-2.7)
Traction, rigid locking nail (n = 3)	24.4	2.1 (0.5-9.3)	2.0	0.5 (0.1-1.3)	23.0	0.9 (0.6-1.4)	6.5	0.9 (0.5-1.6)
Traction, elastic stable nailing (ESIN) (n = 7)	18.1	3.4 (1.2-9.5)	4.9	1.7 (1.0-3.0)	21.9	1.6 (1.2-2.2)	9.1	2.0 (1.4-3.0)
*P*-value^b^	0.18		0.24		<0.001		<0.001	

CI = confidence interval

a Adjusted for age, sex, complications, and fracture type. Mean difference estimated from linear regression models and relative rates estimated from negative binomial models.

b Calculated from the likelihood ratio test comparing models including and not including treating surgeon.

Other treatment types included hip screws, Kirschner wire fixation, Pavlik harness, and single leg casting.

Thirteen patients (8.1%) were noted to have a complication (Table 2). The average time until patients presented with complications was 83.6 days (range zero to 486). Thirty-five total patients (21.9%) were documented to have leg-length discrepancy during this study, determined by the treating surgeon reporting leg-length discrepancy in patient clinic notes. Leg-length discrepancy was not defined by study parameters; surgeons identified leg-length discrepancies ranging from 5 to 36 mm. All cases of leg-length discrepancy resolved spontaneously or became minimal enough so as to not require additional intervention, with the exception of one patient who required limb lengthening using distraction osteogenesis with a hexapod circular external fixator. Nine patients (5.6%) were documented with hardware irritation, considered an expected sequela. The average time until patients presented with expected sequelae was 209.5 days (range 2 to 1,297). The presence of complications during treatment was found to increase the follow-up time by an average of 1.7 months (95% confidence interval = 1.3 to 2.4).

## Discussion

The primary aim of this study was to assess variability in follow-up practices between surgeons at a single center. Duration and frequency of follow-up after pediatric femur fractures varied greatly. Variations in follow-up were markedly associated with the treating surgeon. Through multivariable regression analysis, variations in follow-up could not be completely explained by fracture type, age, or method of fixation. Both the duration and frequency of follow-up including radiographic appointments were found to be associated with the treating surgeon. This suggests that patients receive variations in care depending on whom they are seen and treated by upon injury presentation. Although there was a large range in average duration of follow-up between differing treatment options, the treatment type was not found to be statistically significant in determining the length of time that patients are followed for. We found that after adjustment for the presence of complications, age, sex, and fracture type, the treating surgeon was associated with a variety of outcomes. This suggests that surgeon preference likely plays a role in determining the duration of follow-up in this patient population.

There are currently no comprehensive clinical guidelines standardizing the management and follow-up practices of pediatric femoral fractures. As such, at this institution, there are no standard protocols followed; however, conventional teaching is to follow femur fractures in skeletally immature patients out at least 2 years because of the risk of femoral overgrowth for femoral shaft fractures and potential growth arrest for fractures at or near the epiphyseal plate. Patients included in this series were all discharged from care; thus, surgeons intentionally released patients from the follow-up earlier than conventional recommendations. With the average duration of follow-up time in this study being approximately 10 months, this further shows that surgeons at this institution base follow-up more on clinical judgment than on standard teaching. This in turn leads to the large variability in management that is seen in this study.

Complications and/or expected sequelae were found to increase the duration of time that patients were followed for in this study. The main documented expected sequelae was leg-length discrepancy, with all cases but one resolving or becoming clinically insignificant without the need for additional intervention. The main documented true complications were cast irritation, infection, and notable pain. The average time until complications and expected sequelae arose was approximately 6 months, with the latest documented sequelae being leg-length discrepancy occurring at 43 months after injury. Aside from two patients, all complications occurred within 2 years of the initial injury. This indicates that the current standard teaching of 2-year follow-up is sufficient, although potentially unnecessary, with the overall number of complications remaining low. There was a large range in the duration of time until patients presented with complications, and an overall small number of patients who experienced complications, making it hard to draw definite conclusions. Furthermore, there was no appreciable pattern in which specific complications were presented earlier versus later in treatment.

Because our institution is responsible for specialist pediatric care spanning a wide geographic region, families may be required to travel in from distant locations for follow-up clinic visits. Ideally, patients should be followed long enough to monitor for and identify all potential complications/sequelae while also limiting nonessential patient visits such that time and financial costs could be minimized for families.

This study has a number of limitations. Owing to its retrospective nature, the quality of data collected was dependent on the information available in electronic health records; therefore, certain variables were not available to assess because of inconsistent reporting in patient charts. These include leg dominance, height, weight, medications given in the emergency department and prescribed on discharge, referral to physiotherapy, and casting and implant materials. In addition, the small sample size limited the types of statistical analyses that were suitable to conduct (eg, subgroup analyses were not feasible), particularly when stratifying follow-up data by the surgeon. Most current literature assesses fewer variables, including smaller age ranges, fewer numbers of treatment options, and a fewer numbers of fracture types. This patient cohort included a wide range of fracture patterns and ages, creating the potential for large variability from this alone and making it difficult to draw conclusions about them as a whole. In addition, the list of search terms may not be fully exhaustive, and it is therefore possible that a small number of cases have been missed in this analysis. Finally, the surgeons in this study saw different volumes of patients, and the patients may have differed in ways not measured.

Although this study demonstrates the prevalence of surgeon practice variability, a prospective study examining long-term functionality in this patient population would be ideal to adjust for these limitations. This study should be used as a guide in the creation of such a prospective study with a larger patient cohort to aid in the generalizability of the results. A prospective study would lend to the development of evidence-based clinical guidelines for the management of pediatric femoral fractures to optimize patient care.

Notable variability in follow-up was identified based on the treating surgeon and the type of surgery done. A standardized clinical care guideline for pediatric fractures could reduce nonessential patient visits and ensure that patients are followed sufficiently long to identify expected sequelae and potential complications. This study provides a baseline for the development of additional prospective studies, ultimately culminating in the development of an evidence-based clinical guideline for the management of pediatric femoral fractures.
